# Association between eNOS Gene Polymorphism (T786C and VNTR) and Sickle Cell Disease Patients in Ghana

**DOI:** 10.3390/diseases6040090

**Published:** 2018-09-29

**Authors:** Charles Antwi-Boasiako, Bartholomew Dzudzor, William Kudzi, Alfred Doku, Campbell Andrew Dale, Fredericka Sey, Kate Hgar Otu, Gifty Dankwah Boatemaa, Ivy Ekem, John Ahenkorah, Daniel Gyingiri Achel, Elvis Twumasi Aboagye, Eric S. Donkor

**Affiliations:** 1 Department of Physiology, School of Biomedical and Allied Health Sciences, University of Ghana, Accra +233, Ghana; gdankwah@gmail.com; 2 Department of Medical Biochemistry, School of Biomedical and Allied Health Sciences, University of Ghana, Accra +233, Ghana; bartdzudzor7@gmail.com; 3 Centre for Tropical Clinical Pharmacology and Therapeutics School of Biomedical and Allied Health Sciences, University of Ghana, Accra +233, Ghana; wkudzi@yahoo.com (W.K.); atelvis45@gmail.com (E.T.A.); 4 Department of Internal Medicine, School of Medicine and Dentistry, University of Ghana, Accra +233, Ghana; dokukavin@gmail.com; 5 Center for Cancer and Blood Disorders Children’s National Medical Center George Washington University School of Medicine and Health Sciences, Washington, DC 20052, USA; acampbell@childrensnational.org; 6 Center for Clinical Genetics, Korle-Bu Teaching Hospital, Accra +233, Ghana; fredisey@hotmail.com; 7 Department of Nursing and Midwifery, Greenhills School of Health Sciences, Accra +233, Ghana; kateboasiako@yahoo.co.uk; 8 Department of Haematology, School of Medical Sciences, College of Health and Allied Sciences, University of Cape Coast, Cape Coast +233, Ghana; ekem_ivy@hotmail.com; 9 Department of Anatomy, School of Biomedical and Allied Health Sciences, University of Ghana, Accra +233, Ghana; daajohnny@yahoo.com; 10 Applied Radiation Biology Center, Radiological and Medical Sciences Research, Ghana Atomic Energy Commission, Accra +233, Ghana; gachel@gmail.com; 11 Department of Medical Microbiology, School of Biomedical and Allied Health Sciences, University of Ghana, Accra +233, Ghana; ericsdon@hotmail.com

**Keywords:** eNOS, variants, sickle cell disease complications, allele, genotype

## Abstract

Endothelial nitric oxide synthase (eNOS) variants have been found to be associated with several vascular disorders as well as the pathogenesis of sickle cell disease (SCD) complications such as vaso-occlusive crises (VOC). Studies on eNOS gene variants among SCD patients are rare in Ghana and several other African countries. The current study aimed to determine a possible association between variants of the eNOS gene (variable number of tandem repeats in intron 4 and T786C) in SCD complications among Ghanaian patients. This was a cross-sectional study involving 89 HbSS patients with complications and 46 HbSS patients without complications. Genomic DNA was extracted from leukocytes in the buffy coat and separated from collected whole blood samples of the study participants. PCR amplification, followed by restriction fragment length polymorphism (RFLP) was used to genotype T786C (rs2070744) variants. Variable number of tandem repeats (VNTR) in intron 4 was genotyped by PCR and direct electrophoresis. There was a significant difference in the genotype frequency of the T786C variant between HbSS patients with complications and those without complications (*p* = 0.0165). However, there was no significant difference in the VNTR intron 4 variant of the eNOS gene between patients with complications and those without complications (*p* > 0.05). The study shows an association between the eNOS gene variant (T786C) and complications in SCD.

## 1. Introduction

Sickle cell disease (SCD) patients constitute 1% of the global population, and over 75% of this percentage is found in sub-Saharan Africa [1,2]. In Ghana, the incidence of SCD is about 2% of all births per year [3]. It is a major genetic disease associated with increased mortality in Ghana [4]. The pathophysiology of SCD is believed to be caused by rigid red blood cells resulting from the sickle hemoglobin, which leads to tissue infarction and organ dysfunction in most patients [5]. The most frequently reported symptoms at the instance of morbidity or mortality in hospitals are vaso-occlusive crises (VOC) marked by severe pain in the bones and muscles [6,7]. 

Soluble endothelial cell adhesion molecules (ICAM-1, VCAM-1, and E-selectin) play a vital physiological role in the recruitment and binding of inflammatory cells to vascular endothelium, particularly in venules [8]. The expression of these molecules has been found to be modulated by endothelial nitric oxide (NO) produced by the endothelial nitric oxide synthase (eNOS) gene. Nitric oxide is known to suppress the expression of these adhesion molecules [9]. 

In vivo NO is synthesized during the enzymatic conversion of L-arginine to L-citrulline by three isoforms of the NOS enzyme such as endothelial NOS (NOS1, NOS2, and NOS3) [10]. The most abundant form of these isoforms is eNOS, which is derived from vascular endothelium [11]. This enzyme has been shown to play a very important role in the pathogenesis of VOC in sickle cell transgenic mice [10]. Previous studies have found the eNOS gene to be a critical regulator of vasodilation and an inhibitor of cell adhesion and aggregation, protecting humans and animals from vaso-occlusion [12]. In normal, healthy individuals, the level of NO in the plasma is found to be linked with the haplotypic variation of the eNOS gene [13]. Even though several polymorphic forms of this gene have been found, the most important polymorphic variants of the eNOS gene which are of clinical importance and are responsible for bringing about variations in NO levels are the T786C variant in the promoter region, the Glu298Asp variant in exon 7, and the variable number of tandem repeats (VNTR) in intron 4 [10]. These eNOS variants have been found to be associated with several vascular disorders such as myocardial infarction [14], atherothrombosis [15], erectile dysfunction [16], stroke [17], and renal disease [18] in the healthy, general population of Asian and European origin. Moreover, several studies have suggested the involvement of eNOS variants in the pathogenesis of sickle cell complications such as vaso-occlusive crises and acute chest syndrome [19,20,21,22]. 

Results from previous work conducted in India suggest that the eNOS gene variant is associated with SCD and may act as a genetic modifier of phenotypic variation in these patients [12]. Another study conducted in Africa, however, reported that eNOS variants are less frequent in SCD patients and that they lack any functional significance among these patients [23]. Evidence from the study of Thakur et al. [23] indicates that endothelin-1, rather than the eNOS gene variant, is associated with SCD in Africa. A recent study done by Navaro et al. [24] confirms an earlier report by Thakur et al. [23] and Nada [25] who demonstrated that the eNOS gene polymorphism is not associated with SCD. Thus, findings from these studies suggest that the possible association between eNOS gene variants and SCD seem to be related to ethnogenomic diversity, among other factors. In Ghana, studies have been done on the role of NO in SCD [26], as well as the epidemiology of SCD [27]. There is currently no information on the possible association of the eNOS gene variant among SCD patients in Ghana. The current study aimed to investigate the association of eNOS gene variants (T786C and VNTR) in SCD complications in Ghana. 

## 2. Methods

### 2.1. Study Site and Sampling

This study was conducted at Korle-Bu Teaching Hospital (KBTH), which is the premier health care facility in Ghana and a major referral health facility in Southern Ghana. The hospital hosts the Ghana Institute of Clinical Genetics (GICG), which runs a sickle cell disease clinic daily from Monday to Friday for patients aged 13 years and older. This was a cross-sectional study involving 88 HbSS patients with complications and 46 HbSS patients without complications who were sampled at the sickle cell clinic of GICG. The sickling status of the study participants was confirmed by hemoglobin electrophoresis. Using a standard data collection sheet, information on the demographic and clinical profile of the study participants was recorded. Complications associated with the SCD patients recruited into the study included those with vaso-occlusive crisis, leg ulcers, and priapism. Vaso-occlusive crisis was clinically defined as pains in the bones, muscles and joints that is not attributable to any other cause and requiring parenteral analgesia and admission into the sickle cell clinic [28]. Leg ulcers were also defined as defects in the skin immediately below the level of the knee and above the foot persisting for six or more weeks [28,29]. SCD patients with leg ulcers recruited in the current study were not necessarily active. Priapism was defined as a purposeless, persistent penile erection which is not accompanied by sexual desire or stimulation, lasting more than 6 h [30]. 

Ethical approval was obtained from the Ethical and Protocol Review Committee of the University of Ghana Medical School, and informed consent was obtained from all the study participants.

### 2.2. DNA Extraction and Genotyping

Genomic DNA was extracted from leukocytes in the buffy coat separated from whole blood samples (n = 134) by using the Quick-gDNATM Blood MiniPrep DNA extraction kit (Epigenetics Company, Irvine, CA, USA) according to the manufacturers’ protocol. Isolated DNA concentration and purity was determined with NanoDrop 2000/2000C (Thermo Scientific, Boston, MA, USA). Polymerase chain reaction (PCR) amplification followed by direct electrophoresis was used to genotype variable number of tandem repeats (VNTR) intron 4 (27 bp TR), while RFLP was used to genotype T786C (rs2070744) variants as previously described by Thomas et al. [31] with sequence-specific primers. 

#### 2.2.1. Genotyping of VNTR 27 bp Intron 4 of eNOS Gene

The extracted DNA was amplified for polymorphic VNTR in intron 4 by PCR with a standard protocol [29] and previously published primers [28]. Primer sequences 5′-CTATGGTAGTGCCTTGGCTGGAGG-3′ (forward) and 5′-ACCGCCCAGGGAACTCCGCT-3′ (reverse) were used for the PCR amplification. The reaction was performed for 35 cycles in a Gene Pro thermal cycler (HanghouBioer Technology Co., Ltd., Hangzhou, China). The conditions for the PCR were as follows: initial denaturation was done at 94 °C for 4 min, denaturation at 94 °C for 1 min, annealing at 63 °C for 30 s, extension at 72 °C for 5 min, and final extension at 72 °C for 5 min. The products were then resolved on 3% agarose gel for VNTR classification; 4a, 4b, 4c, 4d. Fragments of 169, 196, 223, and 142 bp correspond to eNOS alleles 4a, 4b, 4c, and 4d respectively, and they were estimated with a TriDye 100 bp DNA ladder (New England Biolabs Inc., Ipswich, MA, USA).

#### 2.2.2. Genotyping of T786C (rs2070744) Variation in eNOS Gene Promoter Region

PCR-RFLP was used to genotype the T786C (rs2070744) variation with the primer pair 5′-TGGAGAGTGCTGGTGTACCCCA-3′ and 5’-GCCTCCACCCCCACCCTGTC-3′. The reaction was performed for 35 cycles in a Gene Pro thermal cycler (HanghouBioer Technology Co., Ltd., Hangzhou, China). The PCR mixtures were heated to 94 °C for 4 min for denaturation and underwent 35 cycles at 94 °C for 30 s for denaturation, 65 °C for 30 s for annealing, and 72 °C for 1 min for extension. Finally, extension was conducted at 72 °C for 5 min. The product from the PCR reactions was digested with the MspI restriction enzyme for genotype calling. The eNOS variant contains a unique MspI restriction site, fragment sizes producing fragments of 140 and 40 base pairs for the wild-type (T) allele, or 90, 50, and 40 base pairs for variant (C). The size of amplified PCR products and digested fragments were estimated with a TriDye 100 bp DNA ladder (New England Biolabs Inc., Ipswich, MA, USA).

### 2.3. Statistical Analysis

Data from the study was entered into MS-EXCEL and was analyzed using SPSS version 20.0 software. The differences between the two groups of subjects (HbSS patients with complications and HbSS patients without complications) were analyzed statistically using the student *t*-test for unpaired data. Analysis of variance (ANOVA) was used to compare the difference between more than two means of groups of subjects for normally distributed data. A multinomial logistical analysis was performed to investigate the association between the eNOS gene variant and SCD complications. Statistical significance was defined as *p* < 0.05. 

## 3. Results

Of the SCD patients recruited, 46 were without complications and 88 were with complications. A total of 56 HbSS VOC, 21 HbSS leg ulcers, and 11 with HbSS priapism comprised those with complications. The mean age of the study participants was found to be 25.5 ± 9.7 years for SCD patients with complications and 31.9 ± 10.0 years for those without complications; there was no significant difference between the ages of the two groups of study participants (*p* > 0.05). There was a significant difference in the genotype frequency of the T786C variant between HbSS patients with complications and those without complications (*p* < 0.05). A majority (62.2%) of patients with HbSS without complications had the CC genotype of the T786C variant. The study did not find a significant difference in the VNTR intron 4 variant of the eNOS gene between patients with complications and those without complications (*p* > 0.05). The 4d allele was found only in SCD patients who presented with complications (Table 1). The allele frequencies were not significantly different in HbSS patients with complications and those without complications (*p* > 0.05) (Table 2).

As reported in Table 3, SCD patients with the TC and CC variant had a high risk of developing leg ulcers (OR, 10.33; 95% CI, 1.24–86.06) and (OR, 10.38; 95%CI, 1.781–60.47) respectively. 

## 4. Discussion

The eNOS gene has been screened for variations in different populations [12,32,33], and previous reports have indicated a substantial interethnic diversity in the distribution of the polymorphic forms of this gene [31,34]. In this study, we investigated eNOS variants among SCD patients for the first time in Ghana. The genotype frequency of T786C of the eNOS gene was found to be significantly higher (*p* = 0.0165) in SCD patients with complications in the current study. This suggests that eNOS gene variants may be associated with SCD complications in Ghana as has been reported among other populations of SCD patients elsewhere [12]. In Central India, a similar study reported that SCD patients have significantly higher frequencies of heterozygous and homozygous variant genotypes of the T786C eNOS gene and low levels of plasma nitrite (NO_2_) [12]. In that study, the SCD severe group had significantly lower levels of plasma NO_2_ and higher frequencies of genotypes of the eNOS gene in contrast to the SCD mild group of patients [12]. Our data, however, contrasts with other studies in Mali [23] and the United States (among African Americans) [24] that indicated that eNOS expression has no significance in SCD. Indeed, ethnicity could be an important factor that possibly accounted for the differences between previous studies and the current study [31,34]. Although the eNOS T786C variant has been described as a genetic risk factor for acute chest syndrome in adult female SCD patients [35], findings from our study suggest a possible link with other complications of SCD such as leg ulcers. 

While the eNOS VNTR is associated with the mean plasma nitric oxide (NO) level [13], the eNOS C-786 variant reduces gene promotor activity in SCD patients [35,36], leading to endothelial dysfunction and reduced NO production in the vascular endothelium [37]. Hence, VNTR variants of the eNOS gene might contribute to the overall expression of plasma NO levels, which are responsible for maintaining normal vasomotor tone, and pathogenesis of sickle cell complications in SCD patients. In addition, eNOS has been shown to play an important role in endothelial dysfunction. A variation of this gene has also been implicated in the pathogenesis of sickle cell complications such as vaso-occlusive crises and acute chest syndrome [19,20,21,22]. The VNTR variant was, however, not significantly different among SCD complications and those without complications in the current study. Intron 4 alleles eNOS4a, eNOS4b, eNOS4c, and eNOS4d have been studied in different populations and under different conditions to determine their possible physiologic and/or disease implication. Such population studies include healthy controls as well as patients with acute chest syndrome, venous thromboembolisms, preeclampsia, myocardial infarction, and Alzheimer’s disease [22,34,35,36,37,38,39,40,41,42,43]. Findings from the current study on the distribution of intron 4 alleles are indeed consistent with a previous work conducted among Africans, African Americans, and Caucasians in the United States of America [31]. They reported that the 4c and 4d alleles are less frequently encountered with minimal distribution, particularly in the African population [31]. Of all the intron 4 alleles, the 4c and 4d alleles were found to be less frequent in the present study. It is worth mentioning that all the participants recruited in the current study were ethnicity-matched Africans. The 4d allele was only found in the SCD patients with complications in our current study. If we attribute the distribution of eNOS gene variants to ethnogenomic or interethnic diversity as implicated in the study of Thomas et al. [31], who found the 4d VNTR allele (in intron 4) only in an African population, then we can suggest that the 4d allele, although rarely encountered, can be found mainly among people from Africa or those with an African descent or ancestry, as observed in the current study. The present study, however, did not recruit patients with a non-African heritage. The eNOS intron 4 gene variant is related to endothelial dysfunction and vasculopathy in SCD and could be used to predict an increased susceptibility to vascular complications [44]. Variations in the same gene could be implicated in a large population of other complications of SCD. 

The main limitation of this current study was the small sample size of SCD patients, as well as the inability to recruit other acute complications of SCD. Nevertheless, this study adds to the current literature of SCD genetics, which is scarce in Africa and particularly in Ghana where SCD carries a huge burden. 

## 5. Conclusions

Complications in Ghanaian SCD patients are associated with the eNOS gene variant (T786C) but not the VNTR intron 4 allele. Further studies are needed to address the role of the Glu298Asp variant of the eNOS gene in complications among SCD patients as it was not investigated in this study. 

## Figures and Tables

**Table 1:** Genotypic frequencies for eNOS variants in HbSS patients with complications and those without complications.

Variants	Genotype	HbSS with Complications n (%)	HbSS without Complications n (%)	
T786C (rs2070744)	TT	7 (8.1)	5 (11.1)	χ^2^ = 8.178 *p* = 0.0165
	TC	35 (40.7)	12 (26.7)	
	CC	44 (51.2)	28 (62.2)	
Total		86	45	
Intron 4 (27-bp TR)	4aa	41 (46.6)	21 (45.7)	
	4bb	37 (42.0)	17 (37.0)	χ^2^ = 2.988
	4cc	8 (9.1)	8 (17.4)	*p* = 0.3934
	4dd	2 (2.3)	0 (0.0)	
Total		88	46	

n = number or frequency; SCD = sickle cell disease; *p* < 0.05 was considered statistically significant; eNOS = endothelial nitric oxide synthase.

**Table 2:** Allele frequencies for eNOS variants in HbSS patients with complications and those without complication.

Variants	Allele	HbSS with Complications n (%)	HbSS without Complications n (%)	
T786C (rs2070744)	T	49 (28.5)	22 (24.4)	χ^2^ = 0.4891
	C	123 (71.5)	68 (75.6)	*p* = 0.4843
Total		172	90	
Intron 4 (27-bp TR)	4a	82 (46.6)	42 (45.6)	χ^2^ = 5.977 *p* = 0.1127
	4b	74 (42.0)	34 (37.0)	
	4c	16 (9.1)	16 (17.4)	
	4d	4 (2.3)	0 (0.0)	
Total		176	92	

n = number or frequency; SCD = sickle cell disease; *p* < 0.05 was considered statistically significant; eNOS = endothelial nitric oxide synthase.

**Table 3:** Association of eNOS gene variants in the studied population.

Genotype	HbSS without Complications	HbSS VOC	HbSS Leg Ulcer	HbSS Priapism
	n = 46	n = 56	n = 21	n = 11
	OR (CI)	OR (CI)	OR (CI)	OR (CI)
TT	2.38 (0.35–46.17)	1.12 (0.03–18.21)	1.34 (2.34–57.02)	0.60 (0.03–10.18)
TC	3.22 (0.39–26.07)	2.13 (0.33–13.97)	10.33 (1.24–86.06) *	0.300 (0.01–11.13)
CC	1.35 (0.237–7.72)	1.82 (0.35–9.38)	10.38 (1.781–60.47) *	0.79 (0.03–26.69)
VNTR 4aa	2.19 (<0.001->10)	0.46 (<0.001->10)	5.611 (<0.001->10)	1.49 (<0.001->10)
VNTR 4bb	1.02 (<0.001->10)	0.36 (<0.001->10)	1.059 (<0.001->10)	4.15 (<0.001->10)
VNTR 4cc	0.29 (<0.001->10)	0.10 (<0.001->10)	0.119 (<0.001->10)	0.22 (<0.001->10)
VNTR 4dd	-	-	-	-

* significant, OR = Odds Ratio, CI = 95% confidence interval; eNOS = endothelial nitric oxide synthase.
